# Sex-Dependent Effects of the *APOE* ɛ4 Allele on Behavioral Traits and White Matter Structures in Young Adults

**DOI:** 10.1093/cercor/bhaa251

**Published:** 2020-09-21

**Authors:** Hikaru Takeuchi, Hiroaki Tomita, Ryan Browne, Yasuyuki Taki, Yoshie Kikuchi, Chiaki Ono, Zhiqian Yu, Rui Nouchi, Ryoichi Yokoyama, Yuka Kotozaki, Seishu Nakagawa, Atsushi Sekiguchi, Kunio Iizuka, Sugiko Hanawa, Tsuyoshi Araki, Carlos Makoto Miyauchi, Kohei Sakaki, Takayuki Nozawa, Shigeyuki Ikeda, Susumu Yokota, Daniele Magistro, Yuko Sassa, Ryuta Kawashima

**Affiliations:** Division of Developmental Cognitive Neuroscience, Institute of Development, Aging and Cancer, Tohoku University, Sendai 980-8575, Japan; Department of Psychiatry, Graduate School of Medicine, Tohoku University, Sendai 980-8575, Japan; Department of Disaster Psychiatry, International Research Institute of Disaster Science, Tohoku University, Sendai 980-8575, Japan; Department of Advanced Brain Science, Institute of Development, Aging and Cancer, Tohoku University, Sendai 980-8575, Japan; Division of Medical Neuroimaging Analysis, Department of Community Medical Supports, Tohoku Medical Megabank Organization, Tohoku University, Sendai 980-8575, Japan; Department of Radiology and Nuclear Medicine, Institute of Development, Aging and Cancer, Tohoku University, Sendai 980-8575, Japan; Department of Disaster Psychiatry, International Research Institute of Disaster Science, Tohoku University, Sendai 980-8575, Japan; Department of Disaster Psychiatry, International Research Institute of Disaster Science, Tohoku University, Sendai 980-8575, Japan; Department of Disaster Psychiatry, International Research Institute of Disaster Science, Tohoku University, Sendai 980-8575, Japan; Department of Advanced Brain Science, Institute of Development, Aging and Cancer, Tohoku University, Sendai 980-8575, Japan; Department of Cognitive Health Science, IDAC, Tohoku University, Sendai 980-8575, Japan; Smart Aging Research Center, Tohoku University, Sendai 980-8575, Japan; School of Medicine, Kobe University, Kobe 650-0017, Japan; Division of Clinical research, Medical-Industry Translational Research Center, Fukushima Medical University School of Medicine, Fukushima 960-1925, Japan; Department of Human Brain Science, Institute of Development, Aging and Cancer, Tohoku University, Sendai 980-8575, Japan; Division of Psychiatry, Tohoku Medical and Pharmaceutical University, Sendai 983-8536, Japan; Division of Medical Neuroimaging Analysis, Department of Community Medical Supports, Tohoku Medical Megabank Organization, Tohoku University, Sendai 980-8575, Japan; Department of Behavioral Medicine, National Institute of Mental Health, National Center of Neurology and Psychiatry, Tokyo 187-8553, Japan; Department of Psychiatry, Graduate School of Medicine, Tohoku University, Sendai 980-8575, Japan; Department of Human Brain Science, Institute of Development, Aging and Cancer, Tohoku University, Sendai 980-8575, Japan; ADVANTAGE Risk Management Co., Ltd. , Tokyo 153-0051, Japan; Department of Advanced Brain Science, Institute of Development, Aging and Cancer, Tohoku University, Sendai 980-8575, Japan; Department of Advanced Brain Science, Institute of Development, Aging and Cancer, Tohoku University, Sendai 980-8575, Japan; Research Institute for the Earth Inclusive Sensing, Tokyo Institute of Technology, Tokyo 152-8550, Japan; Department of Ubiquitous Sensing, Institute of Development, Aging and Cancer, Tohoku University, Sendai 980-8575, Japan; Division for Experimental Natural Science, Faculty of Arts and Science, Kyushu University, Fukuoka 819-0385, Japan; Department of Sport Science, School of Science and Technology, Nottingham Trent University, Nottingham NG11 8NS, UK; Division of Developmental Cognitive Neuroscience, Institute of Development, Aging and Cancer, Tohoku University, Sendai 980-8575, Japan; Division of Developmental Cognitive Neuroscience, Institute of Development, Aging and Cancer, Tohoku University, Sendai 980-8575, Japan; Department of Advanced Brain Science, Institute of Development, Aging and Cancer, Tohoku University, Sendai 980-8575, Japan; Department of Ubiquitous Sensing, Institute of Development, Aging and Cancer, Tohoku University, Sendai 980-8575, Japan

**Keywords:** *APOE* genotype, imaging genetics, sex interaction effects, white matter structures, young adults

## Abstract

The *APOE* ɛ4 allele is associated with a risk of Alzheimer’s disease in the elderly, with the association being pronounced in females. Conversely, findings of the effects of the *APOE* ɛ4 allele in young adults are mixed. Here, we investigated the sex–genotype interaction effects of the *APOE* ɛ4 allele on cognitive functions as well as brain structures among 1258 young adults. After adjusting for multiple comparisons, there were significant effects of the interaction between sex and the number of *APOE* ɛ4 allele on some speed tasks (e.g., simple processing speed tasks and the reverse Stroop task) as well as on regional white matter volume (rWMV). The observed sex–genotype interaction conferred better cognitive performance and greater rWMV in the anterior frontal and precentral white matter areas in females having more *APOE* ɛ4 alleles and reduced rWMV in the same areas in male having more *APOE* ɛ4 alleles. These findings support the long-debated antagonistic pleiotropic effects of the *APOE* ɛ4 allele in females.

## Introduction

Apolipoprotein E (APOE) is a serum protein as well as a major constituent of chylomicrons, very-low-density lipoprotein, and some subforms of high-density lipoprotein. It is involved in the transport of cholesterol and fatty acids as well as neuroplasticity and neural repair (Boyles et al. 1989). It is encoded by *APOE*, which has three common alleles (ɛ2, ɛ3, and ɛ4) (Das et al. 1985). Several studies have shown that the *APOE* ɛ4 allele is a vital risk factor of Alzheimer’s disease in the elderly, with this association being particularly higher in females than in males during the earlier old age (Neu et al. 2017). Additionally, this allele has a relationship with lower cognitive functions in healthy elderly individuals, and some studies have indicated stronger effects in females than in males (Beydoun et al. 2012), although this finding has not been evaluated by meta-analyses. This type of *APOE* ɛ4−sex interaction has been shown in a biomarker that distinguishes Alzheimer’s disease (tau pathology), and stronger relationships between *APOE* ɛ4 allele and greater values in biomarkers of Alzheimer’s disease are observed in females (Altmann et al. 2014). This *APOE* ɛ4−sex interaction might be moderated by estrogen because one study reported that females with the *APOE* ɛ4 allele who underwent estrogen replacement therapy had a reduced risk of Alzheimer’s disease (Rippon et al. 2006).

It is noteworthy that some studies have indicated that the *APOE* ɛ4 allele has a relationship with better cognitive performance in young adults, particularly in tasks that need attention instead of those that require memory (for reviews, see Han and Bondi 2008; Rusted and Carare 2015). With the existing literature in mind, an “antagonistic pleiotropy” hypothesis has been suggested, wherein the *APOE* ɛ4 allele has differential effects on cognitive performance, depending on age (Han and Bondi 2008). Nonetheless, a recent meta-analysis of the effects of the *APOE* ɛ4 allele on cognitive functions in young adults has demonstrated that there is a trend of improved executive functions among young adults and no other differences in other areas across both males and females (Weissberger et al. 2018).

Several neuroimaging studies have assessed the effects of the *APOE* ɛ4 allele on brain structure among healthy people. Numerous such studies in healthy elderly individuals have indicated that this allele has a relationship with greater atrophy (as shown by lower regional brain volumes as well as deficits in other neuroimaging measures) (Cherbuin et al. 2007). Moreover, some of these studies have described stronger effects in females (Sampedro et al. 2015). However, structural studies, including young adults, have shown mixed effects of the *APOE* ɛ4 allele. These studies have particularly focused on the medial temporal lobe structures. A smaller-scale study has described the *APOE* ɛ4 allele’s relationship with a lower quantitative index (cortical thickness) in the medial temporal lobe (Shaw et al. 2007), whereas a recent study with a large sample size has shown that there are no relationships between *APOE* genotypes and quantitative index (volume) in the medial temporal lobe (Khan et al. 2014). Another study that included a small sample size has described the relationships between *APOE* genotypes and white matter structures (Heise et al. 2011).

Briefly, there have been various studies on the effects of *APOE* genotypes. In studies of the elderly, the main effects of *APOE* genotypes (regardless of sex) as well as the effects of interaction between sex and *APOE* genotypes on the risk of Alzheimer’s disease, cognitive functions, and neural mechanisms were explored. In studies of young adults, basically, the main effects of *APOE* genotypes (regardless of sex) on both cognitive functions and neural mechanisms were also explored.

Even with a great number of studies, whether *APOE* genotypes differentially affect both cognitive and neural mechanisms in young males and females (who show fully increased estrogen levels) has not been explored in a large sample, which detects subtle interaction effects. The aim of this study is to address this issue using a large sample. Considering the robust interaction of *APOE* genotypes with sex in the elderly, we have hypothesized the effects of the interaction between APOE genotypes and sex in young adults as well. Because there is a strong genetic association between *APOE* genotypes and Alzheimer’s disease, it is important to have an understanding on how genotypes interact with sex to affect individual cognitive functions as well as the underlying neural mechanisms.

## Material and Methods

### Subjects

The present study is involved in a current project, aiming to explore the relationships among brain imaging metrics, cognitive functions, and a variety of modifying factors (including genetic factors). The present study included 1258 healthy, right-handed subjects (741 males and 517 females) with sufficient data to explore the influence of *APOE* genotypes using whole-brain voxel-based morphometry (VBM). The mean age of the subjects was 20.7 years (standard deviation, 1.8; range, 18–27 years). The subjects included university students, postgraduates, or university graduates for less than 1 year. All subjects had normal vision and had no history of neurological or psychiatric illness. Handedness was assessed using the Edinburgh Handedness Inventory (Oldfield 1971).

For additional details on subjects’ information, see Supplemental Methods. A written informed consent was acquired from all subjects, and this study was approved by the Ethics Committee of the Tohoku University. The descriptions in this study are from our prior study which is in relation to the same project (Takeuchi et al. 2019b).

### Genotyping of Subjects

*APOE* genotype was acquired via a standard protocol using the polymerase chain reaction. *APOE* alleles correspond to allele combinations at single-nucleotide polymorphisms rs429358 and rs7412. The individual genotypes at the two sites were combined to make a single standard *APOE* genotype.

The *APOE* genotype distribution in the 1258 subjects was as follows: ɛ2/ɛ2 (male, *n* = 1; female, *n* = 1, total, *n* = 2, 0.2%), ɛ2/ɛ3 (male, *n* = 57; female, *n* = 38; total, *n* = 95, 7.6%), ɛ3/ɛ3 (male, *n* = 547; female, *n* = 363; total, *n* = 910, 72.3%), ɛ3/ɛ4 (male, *n* = 129; female, *n* = 110; total, *n* = 239, 19.0%), and ɛ4/ɛ4 (male, *n* = 7; female, *n* = 5; total, *n* = 12, 1.0%). Subjects with an undetermined genotype, whether ɛ1/ɛ3 or ɛ2/ɛ4, and subjects with an undetermined genotype for other reasons were not part of this study. Thus, in this study, there are no subjects having a genotype of ɛ1/ɛ3 or ɛ2/ɛ4. The frequency of approximately 1.0% of the ɛ4/ɛ4 genotype is the same as that of the prior study with a young Japanese huge sample (Katsuya et al. 2002). Weinberg equilibrium has shown no deviations from the expected genotype distribution (*P* > 0.05).

In this study, we have divided the subjects based on the number of ɛ4 alleles (0, 1, 2) in accordance with the additive effects model. This strategy is the same with previous larger-scale studies (*N* > 1000) (Acevedo et al. 2010) and is consistent with the finding that the risk of Alzheimer’s disease obviously increased with the increase in the number of ɛ4 allele (Neu et al. 2017). However, other studies have used other models (Cherbuin et al. 2007). For results and notes regarding the model that compared the subjects with ɛ4 carriers and non-ɛ4 carriers as well as the model which added the covariate of the existence of ɛ2 allele (there were only 2 subjects with 2 ɛ2 alleles; thus, the covariate evaluated only whether subjects had at least one allele) in addition to the number of ɛ4 alleles (0, 1, 2), see the Supplemental Methods section. Basically, using these other models did not change the overall conclusion of the study. Comparisons between subjects with ɛ2/ɛ2 and ɛ2/ɛ3 genotypes were also indicated in the Supplemental Methods, Supplemental Results, and Supplemental Discussion sections.

### Psychological Measures

Subjects underwent the following neuropsychological tests. The descriptions in this subsection are from the prior study that employed the same methods (Takeuchi et al. 2015).

[A] A (computerized) digit span task for the evaluation of working memory (Takeuchi et al. 2011).

[B] Raven’s advanced progressive matrix test (Raven 1998): a nonverbal reasoning task and a representative measure of general intelligence.

[C] Tanaka B-type intelligence test (TBIT) (Tanaka et al. 2003): a nonverbal mass intelligence test used for third-year junior high school and older examinees. This test determines psychometric intelligence based on multiple forms of speed tasks. It does not include story problems but uses figures, single numbers, and letters as stimuli. In all the subtests, the subjects have to solve as many problems as possible within a certain period (a few minutes) of time. For details on these subtests, see Takeuchi et al. (2013a).

[D]: The perception factor of TBIT: the subscore focusing on simple processing speed (PS).

[E] Word-Color and Color-Word tasks: the measures of simple PS.

[F] Reverse Stroop task and Stroop task: the measures of the executive function or inhibition. Tasks [E] and [F] are part of the Hakoda’s version of the Stroop task (matching-type). Unlike the traditional oral naming Stroop task, Hakoda’s version is a matching-type Stroop task that requires subjects to check whether the answers that they have chosen are right. The test comprises two control tasks (Word-Color and Color-Word): a Stroop task and reverse Stroop task. For details on these subtests, see Takeuchi et al. (2014).

[G] The S-A creativity test: a measure for creativity by divergent thinking. Subjects have to create as many notions as possible to the given tasks within 5 min. For details, see our previous study (Takeuchi et al. 2010).

In various periods of the projects, somewhat different sets of psychological measures were collected from subjects because of the limited time for the experiment and the temporal focus on specific study purposes, leading to a number of subjects in each analysis as shown in Table 1.

**Table 1:** Psychometric scale scores for each number of *APOE* ɛ4 alleles with statistical values

	ɛ4 = 0 (male) (*N* = 605) (20.8 ± 1.9 y.o.)	ɛ4 = 1 (male) (*N* = 129) (20.9 ± 1.9 y.o.)	ɛ4 = 2 (male) (*N* = 7) (19.6 ± 1.5 y.o.)	ɛ4 = 0 (female) (*N* = 402) (20.5 ± 1.5 y.o.)	ɛ4 = 1 (female) (*N* = 110) (20.8 ± 1.6 y.o.)	ɛ4 = 2 (female) (*N* = 5) (21.6 ± 1.9 y.o.)	Main effect *P* value^a^ (uncorrected, FDR)	Sex interaction effect^b^ [*P* (uncorrected, FDR), r (males, females)]
Digit span (*N* = 1252)	36.83 ± 6.97	37.55 ± 8.00	29.83 ± 4.67	34.85 ± 6.42	35.19 ± 6.30	34.00 ± 4.82	1.000, 0.758	0.804, 0.732 −2.0 × 10^−4^, 0.014
RAPM (*N* = 1258)	28.81 ± 3.76	28.48 ± 4.13	23.57 ± 3.33	27.98 ± 3.89	28.14 ± 3.56	28.4 ± 2.87	0.097, 0.153	0.765, 0.732 −0.084, 0.019
Total score of TBIT (*N* = 1136)	114.6 ± 12.1	112.6 ± 11.5	107.3 ± 8.3	109.5 ± 11.5	111.0 ± 11.0	115.0 ± 11.9	1.000, 0.758	0.004, 0.014^*^ −0.081, 0.062
Perception factor of TBIT (*N* = 1136)	49.51 ± 7.43	48.64 ± 6.65	45.86 ± 3.52	48.92 ± 6.61	49.80 ± 6.53	52.5 ± 7.53	0.272, 0.338	0.002, 0.009^*^ −0.062, 0.068
Word-Color task (items) (*N* = 1256)	71.25 ± 8.18	70.71 ± 7.37	67.14 ± 5.3	70.00 ± 7.28	72.27 ± 7.19	74.20 ± 6.05	0.211, 0.288	<0.0002^c^, 0.001^*^ −0.043, 0.136
Color-Word task (items) (*N* = 1256)	52.38 ± 7.18	51.39 ± 6.05	50.71 ± 3.37	52.77 ± 6.31	53.85 ± 6.89	55.00 ± 3.41	0.643, 0.732	0.011, 0.030^*^ −0.058, 0.075
Reverse Stroop task (items) (*N* = 1255)	59.90 ± 8.51	59.18 ± 7.66	52.43 ± 6.65	59.54 ± 7.70	61.87 ± 7.93	63.80 ± 8.84	0.804, 0.732	<0.0002^c^, 0.001^*^ −0.066, 0.130
Stroop task (items) (*N* = 1256)	48.34 ± 7.88	47.32 ± 7.75	46.14 ± 3.52	49.63 ± 6.60	49.94 ± 6.25	51.20 ± 3.66	0.046, 0.105	0.101, 0.153 −0.055, 0.027
S-A creativity test (*N* = 1258)	37.18 ± 10.59	37.82 ± 9.97	46.71 ± 11.56	39.07 ± 9.69	39.81 ± 10.71	45.2 ± 10.78	0.059, 0.115	1.000, 0.758 0.057, 0.051

Notes: RAPM, Raven’s Advanced Progressive Matrix

^a^ *P* values for main effects (entire cohort) of the *APOE* ɛ4 allele

^b^ *P* value for the sex and the *APOE* ɛ4 allele interaction

^c^For the calculation of FDR-adjusted *P* values, uncorrected *P* values < 0.0002 were treated as 0.0002 (1/5000, once in 5000 iterations).

### Image Acquisition and Preprocessing

All MRI data were obtained with a 3-T Philips Achieva scanner. High-resolution T1-weighted structural images were acquired for VBM. Diffusion-weighted data were obtained with the use of a spin-echo echo-planar imaging sequence to acquire fractional anisotropy (FA) maps. The parameters for these acquisitions were as described in the prior study and are provided in the Supplemental Methods section (Takeuchi et al. 2013b).

The preprocessing of T1-weighted structural data used the Statistical Parametric Mapping 12 software (SPM12; Wellcome Department of Cognitive Neurology, London, UK) implemented in Matlab (Mathworks Inc., Natick, MA, USA).

Using the new segmentation algorithm and the diffeomorphic anatomical registration through exponentiated Lie algebra (DARTEL) registration process implemented in SPM12, the T1-weighted structural images of each individual were segmented and normalized to the Montreal Neurological Institute (MNI) space to obtain images with 1.5 × 1.5 × 1.5 mm^3^ voxels. In addition, we performed a volume change correction (modulation) (Ashburner and Friston 2000). Subsequently, the generated rGMV and rWMV images were smoothened by convolving them with an isotropic Gaussian kernel of 8-mm full width at half maximum (FWHM). The descriptions in this paragraph were mostly reproduced from our previous study using the exact same methods (Takeuchi et al. 2018).

The preprocessing of the diffusion data was performed using SPM8, which is implemented in Matlab. First, with the use of the prior validated (Takeuchi et al. 2013b) twisted two-step segmentation process, methods of the new segmentation algorithm and DARTEL-based registration process were altered to account for the signal distribution of FA within the white matter, FA maps were normalized, and these normalized FA images were then masked using a custom mask image, which is most likely the white matter and smoothed. Details of these preprocessing procedures as well as their validation were shown in prior study (Takeuchi et al. 2013b). For more detailed descriptions and rationales of these procedures, please see the Supplemental Methods section.

### Statistical Group-Level Analysis of Imaging and Psychological Data

Behavioral data were assessed using the R software, version 4.0.0 (Team-R-Core 2014). The main effects of the *APOE* ɛ4 allele (the number of alleles) on psychological measures among the entire cohort and the interaction between the number of *APOE* ɛ4 alleles and sex were evaluated with the analyses of covariance (ANCOVAs). As seen in these analyses, sex was a fixed factor, and both age and the number of *APOE* ɛ4 alleles (0, 1, and 2) were covariates. *P* values that were not corrected for multiple comparisons were evaluated with permutation (5000 iterations)-ANCOVAs using the lmPerm package (Wheeler 2010) of R because of the relatively small number of subjects with the ɛ4/ɛ4. We used permutation tests because permutation tests are distribution-free and robust to the small sample size in each group (Tanizaki 2006). In psychological analyses, the statistical results having a threshold of *P* < 0.05, which is corrected for false discovery rate (FDR) with the two-stage sharpened method (Benjamini et al., 2006), were statistically significant. This multiple comparison correction method was used in the results of the abovementioned ANCOVAs. Because nine psychological measures were observed, analyses of the main effects and the sex interaction effects had 18 *P* values for multiple comparison correction.

Second-level statistical analyses were conducted using the SPM8 software. In group-level imaging analyses, we tested for the main effects of the number of *APOE* ɛ4 alleles in the entire cohort as well as the interaction effects between the number of *APOE* ɛ4 alleles and sex on regional brain structures.

In the analyses of regional gray matter volume (rGMV) and regional white matter volume (rWMV), only voxels with a signal intensity of >0.05 were part for all the subjects. The analyses of FA were limited to the white matter mask made as previously mentioned.

Whole-brain analyses were performed using voxel-wise ANCOVA with sex difference as a grouping factor which employs the full factorial option of SPM8. All analyses involved the age and the number of *APOE* ɛ4 alleles as covariates. These covariates were modeled so that each had a unique relationship with imaging measures for each sex. In these analyses, the centering option was utilized for centering the interactions. The main effects of the number of *APOE* ɛ4 alleles [contrasts of (the effects of the number of *APOE* ɛ4 alleles for males and females) defined as (1 1) or (−1–1)] and the interaction between sex and the number of *APOE* ɛ4 alleles [contrasts of (the effect of the number of *APOE* ɛ4 alleles for males, the effect of the number of *APOE* ɛ4 alleles for females) defined as (−1 1) or (1–1)] were evaluated using t-contrasts. We have utilized t-contrasts to evaluate the interaction between both males and females. This procedure was used in our previous neuroimaging studies and other studies investigating sex interaction effects (Yamasue et al. 2008; Yang et al. 2014; Takeuchi et al. 2019a, 2019b). However, the statistical design was not identical to those who employed psychological analyses as are the cases of our previous studies (Takeuchi et al. 2019a, 2019b). This is because to our knowledge, neither R nor SPSS can adopt the abovementioned statistical model of sex-interaction analyses employed in neuroimaging analyses using SPM.

A multiple comparison correction was performed with threshold-free cluster enhancement (TFCE) (Smith and Nichols 2009) and randomized (5000 permutations) nonparametric testing with the use of the TFCE toolbox (http://dbm.neuro.uni-jena.de/tfce/). We used a threshold of family-wise error rate which is corrected at *P* < 0.05.

## Results

### Main and Interaction Effects of *APOE* ɛ4 Genotypes on Psychological Metrics

ANCOVAs have demonstrated no significant main effects of *APOE* ɛ4 genotypes (the number of alleles) on psychological test scores, even at the uncorrected level. After correction for multiple comparisons (FDR), ANCOVAs have shown no significant interaction effects between sex and the number of *APOE* ɛ4 alleles on the total TBIT score (a measure of psychometric intelligence using speed tasks), the perception factor of TBIT, Word-Color task, Color-Word task (all measures of simple PS), and reverse Stroop task (a measure of executive function and inhibition). In the case of Word-Color task and reverse Stroop task, the interactions were primarily regulated by a significant positive correlation with the number of *APOE* ɛ4 alleles in females (Word-Color task, *r* = 0.136, *P* = 0.002; reverse Stroop task, *r* = 0.130, *P* = 0.003, simple linear regression analyses) rather than a negative correlation in males (e.g., Word-Color task, *r* = −0.043, *P* = 0.241; reverse Stroop task, *r* = −0.066, *P* = 0.075). However, for other significant results, the strength of negative correlations in males and the positive correlations in females were not substantially different (Table 1).

### Main and Interaction Effects of *APOE* ɛ4 Genotypes on Brain Structures

**Figure 1:**
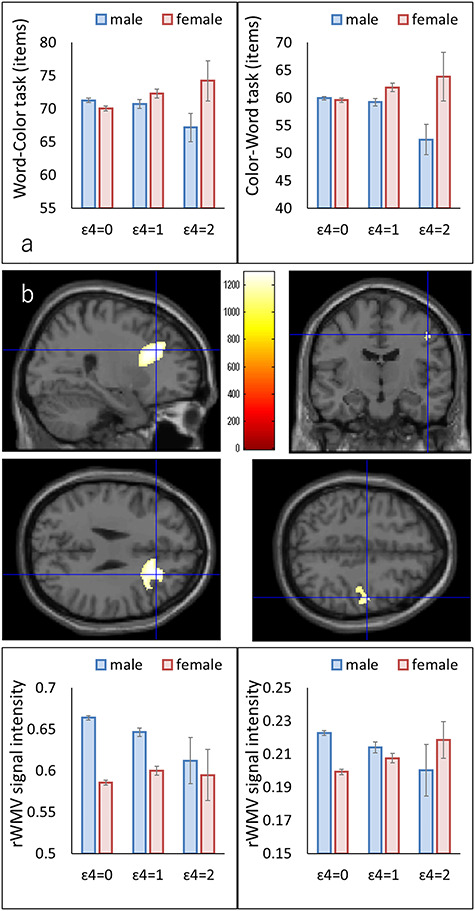
*APOE* ɛ4 genotype−sex interaction effects on cognitive function as well as regional brain volumes in young adults. (a) Effects on cognitive test performance. The left and right panels show the results of the Word-Color task, which is a measure of simple processing speed, and reverse Stroop task, which is a measure of executive function or inhibition, respectively. Error bars constitute the standard errors of mean. (b) Effects on rWMV. The left panels show an rWMV cluster underneath the right lateral prefrontal cortex and close to the anterior cingulate cortex, whereas the right panels show an rWMV cluster below the right precentral gyrus. The upper panels show the areas that are remarkably influenced by the *APOE* ɛ4 allele–sex interaction. The results were acquired using a threshold for TFCE of *P* < 0.05 based on 5000 permutations. Findings are overlaid on a “single-subject T1” image from SPM8. The color bar constitutes the TFCE value. The lowest two panels are the profiles of rWMV in the areas of significant clusters in both males and females of each number of the *APOE* ɛ4 allele.

Whole-brain ANCOVAs of rGMV have shown no significant main effects of *APOE* ɛ4 genotypes and no interaction effect between sex and the number of *APOE* ɛ4 alleles on rGMV. Alternatively, whole-brain ANCOVAs have shown that there is a significant effect of the interaction between sex and *APOE* ɛ4 genotypes on two, broad white matter clusters: one below the right precentral gyrus and the other underneath the right lateral prefrontal cortex/right anterior cingulate cortex, spreading across the genu and body of the corpus callosum, anterior limb of the right internal capsule, right anterior and superior corona radiata, right external capsule, and right superior fronto-occipital fasciculus (Fig. 1b). Within both the clusters, the number of *APOE* ɛ4 alleles have shown that there is a significant negative correlation with mean rWMV in males (the former cluster, *r* = −0.113, *P* = 0.002; the latter cluster, *r* = −0.106, *P* = 0.004; simple linear regression analyses) and a significant positive correlation in females (the former cluster, *r* = 0.097, *P* = 0.028; the latter cluster, *r* = 0.120, *P* = 0.006). The whole-brain ANCOVAs of rWMV have shown that there are no significant main effects of the *APOE* ɛ4 allele on rWMV.

Finally, according to FA’s whole-brain ANCOVAs, there are no significant main effects and no effects of the interaction between sex and the *APOE* ɛ4 allele on imaging measures.

### The Associations between rWMV of Significant Clusters and Significant Behavioral Measures

Post hoc partial correlation analyses have shown that after controlling age, sex, and the *APOE* ɛ4 allele, the rWMV of the previously mentioned significant cluster underneath the right precentral gyrus (cluster No.2 in Table 2) has shown a significant positive correlation with the performance of Word-Color task (*P* = 0.017, partial correlation coefficient = 0.068), performance of Color-Word task (*P* = 0.017, partial correlation coefficient = 0.068), and performance of reverse Stroop task (*P* = 0.037, partial correlation coefficient = 0.059) but not with the total and perception scores of TBIT (*P* > 0.1). Further, post hoc partial correlation analyses have indicated that after controlling age and sex rWMV of another larger significant cluster (cluster No.1 in Table 2), a significant positive correlation with performance of Word-Color task (*P* = 0.008, partial correlation coefficient = 0.074), performance of Color-Word task (*P* = 0.016, partial correlation coefficient = 0.068), and performance of reverse Stroop task (*P* = 0.002, partial correlation coefficient = 0.089) was shown but not with the total and perception score of TBIT (*P* > 0.1).

**Table 2:** Regional brain volumes significantly influenced by the interaction between sex and the *APOE* ɛ4 allele

No	Included large bundles^*^^*^ (number of significant voxels in the left and right side of each anatomical area)	x	y	z	TFCE value	Corrected *P* value (TFCE, FWE)	Cluster size (voxel)
1	The genu of the corpus callosum (54)/the body of the corpus callosum (39)/the anterior limb of the internal capsule (R:75)/the anterior corona radiata (R:674)/the superior corona radiata (R:66)/the external capsule (R:14)/the superior fronto-occipital fasciculus (R:33)	25.5	27	28.5	1294.65	0.032	2071
2	None	48	−12	45	1152.56	0.043	238

Note: ^**^The anatomical labels and significant clusters of major white matter fibers were determined using the ICBM DTI-81 Atlas (http://www.loni.ucla.edu/).

## Discussion

To our knowledge, this is the first study that has assessed the sex-dependent effects of *APOE* genotypes on cognitive functions and regional brain structures using a very large sample of young adults (age during which females show fully increased estrogen levels). Partially consistent with our original hypothesis, the genotype–sex interaction affected the performance of an executive function task (reverse Stroop task) and simple PS tasks as well as an intelligence test, which comprise speed cognitive tasks (total score of TBIT). Specifically, females have indicated a significant positive correlation between the number of *APOE* ɛ4 alleles and performance. Furthermore, partially consistent with our hypothesis, we have seen that there is an effect of the sex–genotype interaction on rWMV just below the right lateral prefrontal cortex and the right anterior cingulate cortex as well as underneath the right precentral gyrus. Precisely, males exhibited significant negative correlations between the number of *APOE* ɛ4 alleles and rWMV, whereas females exhibited significant positive correlations.

Females with a greater number of *APOE* ɛ4 alleles demonstrated superior performance in some speed tasks, which is consistent with the “antagonistic pleiotropy” hypothesis of the *APOE* ɛ4 allele and sex–genotype interaction effects of this gene (in the elderly). These results are also in accordance with the general finding that young *APOE* ɛ4 allele carriers repeatedly exhibit a superior performance, specifically in tasks that require attention rather than memory (Rusted and Carare 2015). Additionally, the effects of the observed sex interaction might explain why previous meta-analyses could not show a strong antagonistic pleiotropic effect of the *APOE* ɛ4 allele in young adults (Weissberger et al. 2018).

The sex–genotype interaction affects rWMV of areas related to attention, executive function, and motor function; therefore, this interaction may underlie the observed behavioral findings. Significant interaction effects were seen in the white matter areas which are underneath the right lateral prefrontal cortex/right anterior cingulate cortex (which also included the genu of the corpus callosum) and underneath the right precentral gyrus. It has been proposed that the anterior cingulate cortex is a part of the task-relevant control of attention and/or response selection in the presence of conflicting information (MacLeod and MacDonald 2000). Conversely, the lateral prefrontal cortex is implied in both executive function and selective attention (Baddeley 2003). The genu of the corpus callosum connects the bilateral lateral prefrontal cortex (Barbas and Pandya 1984) and therefore might mediate interhemispheric information transfer which involves the lateral prefrontal cortex. Together with the white matter areas of the precentral cortex that are a part of motor function, these changed white matter structures might be the basis for some of the behavioral changes that are observed among *APOE* ɛ4 allele carriers (e.g., in simple PS and executive attention tasks). Consistent with this idea, our prior study has indicated that simple PS and Stroop task performance are positively correlated with rWMV of widespread white matter areas (Magistro et al. 2015), and our additional analyses have shown that rWMV of clusters of significant effects of *APOE* ɛ4 allele–sex interaction have a significant positive correlation with the performance of simple PS and Stroop task performance (behavioral measures of significant effects of *APOE* ɛ4 allele–sex interaction). Although it is tempting to speculate that the observed relationships between *APOE* ɛ4 allele and brain structures lead to the relationships between *APOE* ɛ4 allele and functional connectivity that involve relevant regions (such as the hippocampus and the right DLPFC), in the present sample, there are no such associations in supplemental analyses (see Supplemental Methods and Supplemental Results for details). This may be because of the lower associations of resting state fMRI measures with the genotypes (Elliott et al. 2018); however, future studies with a larger sample size may have to further assess this concern.

The cross-sectional study design does not permit the exploration of the precise mechanisms that underlie the *APOE* ɛ4 allele–sex interaction effect among young adults. Here, several possibilities were proposed. First, estrogen and the *APOE* ɛ4 allele may behave synergistically in the elderly. For instance, estrogen regulates the expression of *APOE* (Srivastava et al. 1997), and estradiol promotes synaptic sprouting in response to injury via an *APOE*-dependent mechanism (Stone et al. 1998). Next, the *APOE* genotype–sex interaction in the elderly can be mediated by lacking estrogen levels (Yaffe et al. 2000). Conversely, the mechanisms that underlie the antagonistic pleiotropic effects of the *APOE* ɛ4 allele in young adults are still not clear. *APOE* influences brain activity via multiple mechanisms (Liu et al. 2013). One hypothesis is that *APOE* is not only involved in the clearance of amyloid beta but also in neuronal remodeling. We have seen that young males and females that have greater neuronal plasticity than another gene have shown the evidence of less neural tissues (Takeuchi et al. 2020). This may be partially mediated by the pattern that subjects with the genotype of greater neural plasticity tend to show the signs of less neural tissues with conditions of low exercise levels which are widespread in the modern world (Takeuchi et al. forthcoming). Similar mechanisms might be the cause of low rWMVs in young adults with the *APOE* ɛ4 allele. However, this is highly theoretical, and future studies need to show these mechanisms.

The absence of the effects of the *APOE* ɛ4 allele on brain structure independent of sex is not consistent with the effects that are observed in various prior studies, possibly because of methodological differences, for instance, image preprocessing. For example, a prior study (Heise et al. 2011) exploring FA values among 34 young adults and 37 older adults has reported that the *APOE* ɛ4 allele is related to reduced FA across age groups, whereas the present study with 1258 subjects could not detect the effects of the *APOE* ɛ4 allele on FA in young adults. The small sample size used might lead to several false positives or publication bias (Button et al. 2013). In a similar manner, a small-scale study of young adults has shown that there are significant effects of the *APOE* ɛ4 allele on hippocampal volume (O'Dwyer et al. 2012); however, a study with a large sample size (*N* > 1400) could not show such effects (Khan et al. 2014), which is consistent with the present negative findings that are related to rGMV.

The current study has a number of limitations. First, the focus of the study was on young adults; thus, it is not clear whether or how the *APOE* ɛ4 allele influences cognitive functions in children or middle-aged adults. Although the effects of *APOE* genotypes are firmly established in the elderly, the effects in young adults are still not clear (Rusted and Carare 2015). In fact, even in this study, effects on white matter areas were marginally significant and thus need to be replicated. However, limiting the sample to young adults is also a strength of this study, considering that the age dependence of the effects of the *APOE* genotype could prevent more subtle interactions with sex and other factors from being detected. Additionally, similar to the case of studies on college units, we have been focusing on educated samples. However, we used a large sample and increased statistical power, and the lack of robust simple association between *APOE* genotype and cognitive functions across sexes is similar to the findings of the meta-analysis (Weissberger et al. 2018). Conversely, focusing on the educated sample might decrease the range of cognitive test scores relative to a more representative sample, thereby diminishing the detection of simple relationships among cognition and the genotype across the sexes. Future studies might need to confirm this idea.

This is the first study to assess the effects of interaction between sex and *APOE* genotype, which has a strong relationship with the risk of Alzheimer’s disease in elderly individuals, on cognitive traits, and brain structure in a large sample of young adults. Although this interaction is firmly established in elderly individuals, the effects of the *APOE* ɛ4 allele in younger adults remain controversial, and it has been stressed in a previous review that it is necessary to focus on the effects of interaction between sex and *APOE* genotype in young adults (Rusted and Carare 2015). The current study shows that the *APOE* ɛ4 allele is related to a better performance in speed tasks, including simple PS tasks and executive function tasks as well as enhanced white matter volumes in the anterior frontal and precentral areas in females, whereas it is the opposite in the white matter volumes in males. Collectively, these findings support the antagonistic pleiotropic effects of the *APOE* ɛ4 allele in females.

## Supplementary Material

APOEWMV_supplementalonlinematerial_CC2ndrev_submitted_bhaa251

## Notes

We thank Yuki Yamada for operating the MRI scanner, Haruka Nouchi for conducting the psychological tests, all other assistants for helping with the experiments and the study, and the study participants and all our other colleagues at IDAC, Tohoku University for their support. The authors would like to thank Enago (www.enago.jp) for the English language review. *Conflict of Interest*: None declared.
