# Supplementary material for: Sex-Dependent Effects of the APOE ɛ4 Allele on Behavioral Traits and White Matter Structures in Young Adults
Source: Cereb Cortex. 2020 Sep 21;31(1):672–80. doi: 10.1093/cercor/bhaa251 (PMC7727389; doi:10.1093/cercor/bhaa251)
Supplement: APOEWMV_supplementalonlinematerial_CC2ndrev_submitted_bhaa251 [file apoewmv_supplementalonlinematerial_cc2ndrev_submitted_bhaa251.docx]

**Supplemental online material**

**Supplemental Methods**

**Details of recruitment and exclusion criteria of subjects**

They were recruited using advertisements on bulletin boards at Tohoku University or via email introducing the study. These advertisements and emails specified the unacceptable conditions in individuals with regard to participation in the study such as handedness, the existence of metal in and around the body, claustrophobia, the use of certain drugs, a history of certain psychiatric and neurological diseases, and previous participation in related experiments.

A history of psychiatric and neurological diseases and/or recent drug use was assessed using our laboratory’s routine questionnaire, in which each subject answered questions related to their current or previous experiences of any of the listed diseases and listed drugs that they had recently taken. Drug screening was performed to confirm that the subjects were not taking any illegal psychostimulants or antipsychotic drugs, which was one of the exclusion criteria used during the course of the recruitment. Subjects with exclusion criteria should have been excluded before they came to the lab, but if they came for some reason, they had to go back once it was found that they met an exclusion criterion. Consequently, none had a history of neurological or psychiatric illness. In the course of this experiment, the scans were checked for obvious brain lesions and tumors, but there were no subjects having such obvious lesions or tumors.

These descriptions are mostly obtained from our previously published work (Takeuchi et al. 2017).

**Additional details of subjects**

Some of the subjects were also part of our interventional studies; however, only the psychological data and imaging data which are recorded before intervention were used in these studies (Takeuchi et al. 2014a). Psychological tests and MRI scans for other studies were administered alongside those reported in the current study.

Both the advertisements and emails stated the exclusion criteria for the study: left-handedness; a metal in and around the body; claustrophobia; the use of certain drugs; history of head trauma; psychiatric and neurological diseases; and prior participation in similar experiments. We administered the laboratory’s routine questionnaire to all the potential experimental subjects to evaluate psychiatric illnesses and recent drug use. In this questionnaire, each subject answered questions based on their current or previous experiences and has written down any illnesses and drugs that they had recently taken. Handedness was assessed using the Edinburgh Handedness Inventory (Oldfield 1971). These evaluations were finished when the potential subjects visited the laboratory. If these candidates did not the before-mentioned criteria, they were not included in the study.

Subjects were then told that they should get enough sleep, maintain their normal schedules, eat sufficient breakfast, and consume normal amounts of caffeinated foods and drinks on the day of cognitive tests and MRI scans. In addition to that, the subjects were informed to avoid alcohol the night before the evaluation.

They were recruited using advertisements on bulletin boards at close universities or through email introducing the study or through ads given in the local town paper. Both advertisements and emails included the unacceptable conditions among individuals regarding participation in the study including handedness, metal in and around the body, claustrophobia, the use of certain drugs, a history of certain psychiatric and neurological diseases, and prior participation in alike experiments.

Descriptions in this subsection are mostly reproduced from another study, which relate to the same project with identical methods (Takeuchi et al. 2015).

**Supplemental analyses of the effects of the *APOE* ɛ4 allele on resting-state functional connectivity**

In this manuscript, we mainly assessed the relationship between *APOE* ɛ4 allele and brain structures, although we also supplementarily investigated the relationship of *APOE* ɛ4 allele with the resting-state functional connectivity (RSFC), which involved pertinent brain areas.

Here, procedures reported in our previous study (Takeuchi et al. 2017) were followed, and most of the descriptions in this subsection are from our previous study.

All MRI data acquisition was conducted with a 3-T Philips Achieva scanner. Overall, 34 transaxial gradient-echo images (64 × 64 matrix, TR = 2000 ms, TE = 30 ms, flip angle = 70°, FOV = 24 cm, slice thickness = 3.75 mm) covering the entire brain were acquired using an echo planar sequence for resting-state fMRI analyses. For this scan, 160 functional volumes were obtained while subjects were resting (resulting in a scan length of 5 min 20 s). During the resting-state scanning, the subjects were instructed to keep still with their eyes closed, as motionless as possible, and not to sleep and not to think about anything in particular, as done in previous studies (Damoiseaux et al. 2006; Greicius et al. 2003).

To avoid motions, pads and magic tapes were used and subjects were given thorough instructions to prevent motion during the scan and explanations as to why motions are not preferable as well as the instruction that excessive motion would lead to re-scan.

Before the rsfMRI scan, BOLD images were obtained during the N-back working memory task (Takeuchi et al. 2011). These images were used in the normalization procedures as described below.

The preprocessing of imaging data was performed using SPM8 implemented in MATLAB and SPM8’s extension software Data Processing Assistant for Resting-state fMRI (DPARSF) [part of the toolbox for Data Processing and Analysis of Brain Imaging (DPABI) (http://rfmri.org/dpabi)(Yan et al. 2016)].

Briefly, rsfMRI BOLD images were non-linearly registered to the BOLD image of the N-back working memory task and were then coregistered to the b = 0 image of the diffusion-weighted images (together with rsfMRI BOLD images). These procedures were performed because diffusion tensor images were appropriate for accurate normalization procedures of EPI images because of their similar shapes with other EPI images, but they have more anatomical information. However, blood-oxygen-level dependent (BOLD) images of the N-back working memory task in our project had more similar shapes with DTI images than the rsfMRI BOLD images. b = 0 images were aligned with the FA image and MD map. Subsequently, all images were normalized using a previously validated two-step new segmentation algorithm of diffusion images and the previously validated diffeomorphic anatomical registration through the exponentiated lie algebra-based registration process (Takeuchi et al. 2013) with DTI images. The voxel size of normalized rsfMRI BOLD images was 3.75 × 3.75 × 3.75 mm^3^. For more details about these procedures, the Supplemental Methods section of our prior study should be checked (Takeuchi et al. 2017). From these images, whole-brain, white matter, and cerebrospinal fluid (CSF) masks were created.

The normalized series of BOLD images were processed by DPARSF for individual level analysis. First, 27 nuisance covariates including the mean timecourse of signals from the voxels within the whole brain mask, the mean timecourse of signals from the voxels within the white matter mask, the mean timecourse of signals from the voxels within the CSF mask, and Friston 24 motion parameters. The Friston 24-parameter model (i.e., six head motion parameters, six head motion parameters one time point before, and the 12 corresponding squared items) (Friston et al. 1996) was used to regress out head motion effects. Recent work indicates that regressing out Friston 24-parameters is more effective than other movement correction methods, such as correction for rigid-body using six parameters, derivative 12 parameters, and voxel-specific 12 regressors (Chao-Gan et al. 2013). To further exclude the residual effect of motion on rsfMRI measures, volume-level mean framewise displacement was computed and used at the second-level analyses as covariates (Power et al. 2012). The processed images were spatially smoothed with 7.5-mm FWHM, and the resultant images were masked with the whole brain mask that was created as described above. The smoothing value was 7.5 mm because this value is twice the voxel size of the original image.

After preprocessing, the fMRI data were temporally band-pass filtered (0.01 < f < 0.08 Hz) to reduce low frequency drift and high frequency. For the RSFC analysis, correlation maps were produced by extracting the BOLD time course from the seed regions and computing the correlation coefficient between this time course and the time course from all other brain voxels. In this study, we examined correlations of activity of the right DLPFC, which is near the area of significant interactions in the current study and which has a key role in cognitive functions, and the bilateral hippocampi, which are critical areas for Alzheimer’s disease and the regions of interest in studies on *APOE* genotype. The seed region of the right DLPFC’s definition was the anatomical mask for the right LPFC, as in previous studies (Song et al. 2008; Takeuchi et al. 2014b) and by intersecting BA46, the right middle frontal gyrus, and the gray matter in WFU_PickAtlas (http://fmri.wfubmc.edu/cms/software). This seed ROI is composed of 70 voxels after the reslicement to the normalized space of this study, and it is not a large ROI. The seed regions of the bilateral hippocampi were made using the bilateral hippocampi mask of AAL option in WFU_PickAtlas.

Correlation values were Fisher z-transformed to improve normality. Finally, RSFC maps were acquired by subtracting the mean value within the whole-brain mask and dividing by the standard deviation of the whole-brain mask (Yan et al. 2013).

The second-level whole-brain analyses were performed similar to those of the main text except for the mask used for areas of analyses, which was the whole-brain mask made previously. Moreover, the framewise displacement, calculated above, was added as a covariate as well in a manner that the variable had a common relationship with the imaging measures for each sex.

**Evaluation of other statistical models**

In this manuscript, the subjects were categorized based on the number of ɛ4 alleles (0, 1, 2) in accordance to the additive effects model. This plan is the same as with previous larger-scale studies (N > 1000) (Acevedo et al. 2010). Additionally, studies on the risk of Alzheimer’s disease vividly indicate that the effects of 2 ɛ4 allele are greater than that of the single ɛ4 allele, indicating that the additive model is appropriate (Neu et al. 2017). However, each sex had various subjects with ɛ4/ɛ4; this number is greater than or equal to several prior neuroimaging studies of *APOE* ɛ4 allele (Cherbuin et al. 2007; Heise et al. 2011; Shaw et al. 2007). Additionally, permutation methods in this study were used, preventing the increase of false positives that appear from the small subjects in some of the groups (Tanizaki 2006).

However, previous studies have embraced diverse models (Cherbuin et al. 2007).

First, studies with small sample sizes usually classify them in accordance with the presence/absence of the APOE ɛ4 allele because only a few may have the ɛ4/ɛ4 genotype (Cherbuin et al. 2007; Shaw et al. 2007). In this study, although, there are only several subjects of ɛ4/ɛ4 genotype in each sex, categorizing subjects according to the presence/absence of the APOE ɛ4 allele did not substantially alter our present findings described below. When this statistical design was taken, the significant findings in the analyses of perception scores of Tanaka B-type intelligence test in Table 2 became marginally insignificant after corrections for multiple comparisons (0.5 < P< 0.1, corrected for FDR) and the significant findings of rWMV in the right pre- and post-central gyrus became marginally insignificant (P = 0.066, corrected at the whole brain), other findings such as the significant white matter findings in the anterior frontal areas are still significant. In this supplemental analysis, the ɛ4/ɛ4 genotype was not excluded because none of the 25 previous volumetric studies of neuroimaging of the ɛ4 allele which were mentioned in the third paragraph of the main text or the review by (Cherbuin et al. 2007) employed that procedure (all of the studies either combined the ɛ4/ɛ4 genotype with ɛ3/ɛ4 or separated them, whether or not they had more than some subjects with a ɛ4/ɛ4 genotype) and because subjects with a ɛ4/ɛ4 genotype are apparently distinguished by the marked increase in the risk of Alzheimer’s diseases (Neu et al. 2017) and this is the most vital and representative genotype of this manuscript’s topic.

Alternatively, previous studies with several thousands of subjects have also classified subjects according to the combination of the APOE ɛ2, ɛ3, and ɛ4 alleles. However, these analyses were not performed in this study because there were only two subjects with the ɛ2/ɛ2 genotype; furthermore, the main aim of this study was to evaluate the effects of the interaction between APOE ɛ4 and sex on brain structures and cognitive functions in young adults. Moreover, the model that included the APOE ɛ2 allele was too complex for the number of subjects in this study, and assumptions regarding the differences among young adults with the APOE ɛ2, ɛ3, and ɛ4 alleles were not made because there were no strong findings in young adults in previous studies.

Additionally, although some prior studies compared 3 groups (ɛ2 carriers, carriers of 2 ɛ3 alleles, and ɛ4 carriers, often excluding carriers of ɛ2/ ɛ4), in this study, 2 × 3 interaction analyses (males and females, × ɛ2 carriers, carriers of 2 ɛ3 alleles, and ɛ4 carriers) that included 6 groups are incompatible with permutation analyses (the software could not handle this analysis). However, alternatively, we used the model that incorporated both the regressor of the number of ɛ4 allele and the regressor of the existence of ɛ2 allele in supplemental analyses. The significant findings of ɛ4 allele in this model were the same as that of the previously mentioned model, categorizing subjects according to the presence/absence of the APOE ɛ4 allele (only the results of the perception score of TBIT and rWMV findings of the right pre- and post-central gyrus became marginally insignificant). Therefore, the regression of the effects of ɛ2 allele did not significantly change the findings of the main text.

Moreover, comparisons among subjects with ɛ3/ɛ3 and ɛ2/ɛ3 genotypes are described in the following text.

**Group-level statistical analyses of imaging and psychological data regarding the effects of the *APOE*** ɛ**2 allele**

Even if the main aim of this study was to evaluate the effects of the *APOE* ɛ4 allele in young adults, we have also made a supplemental evaluation regarding the main and sex interaction effects of the *APOE* ɛ2 allele. The *APOE* ɛ2 allele has robust protective effects against Alzheimer’s disease in elderly individuals in comparison with the *APOE* ɛ3 allele; however, these effects are considerably weaker than the risk conferred by the *APOE* ɛ4 allele (in comparison with the *APOE* ɛ3 allele) (Farrer et al. 1997).

The software programs that are reported in the main text were also used for these analyses.

The *APOE* ɛ2 allele effects were evaluated using a comparison between subjects with ɛ3/ɛ3 genotype and those with ɛ2/ɛ3 genotype because those with ɛ1/ɛ3 genotype and those with ɛ2/ɛ4 genotype were not part of this study (see main text) and because only two subjects have the ɛ2/ɛ2 genotype (not included in these supplemental analyses). Thus, only subjects with ɛ2/ɛ3 and ɛ3/ɛ3 genotypes were involved, among them were 57 males with ɛ2/ɛ3, 38 females with ɛ2/ɛ3, 547 males with ɛ3/ɛ3, and 473 females with ɛ3/ɛ3 genotypes.

In non-whole-brain analyses, the main effects of the *APOE* ɛ2 genotype (one allele) and *APOE* ɛ2 genotype–sex interaction effects on cognitive measures were evaluated using the analyses of covariance (ANCOVAs). In these analyses, sex was a permanent factor, and both age and the number of *APOE* ɛ2 alleles (0 or 1) were covariates. The results with a threshold of P < 0.05, which are corrected for false discovery rate using the two-stage sharpened method (Benjamini et al., 2006), were statistically significant. The correction for multiple comparisons using this method was applied to the results of the 11 ANCOVAs. The dependent variables for ANCOVAs are nine cognitive measures recorded in the main text and two imaging measures (the mean rWMV values of the two significant clusters in the main text). As 11 ANCOVAs were performed, analyses for the main effects and sex interaction effects produced 22 p-values for multiple comparison correction.

In the whole-brain analyses, we utilized voxel-wise ANCOVAs with sex difference as a grouping factor (using the full factorial option of SPM8). All analyses used both age and the number of *APOE* ɛ2 alleles (0 or 1) as covariates. The rest of the procedures were described for whole-brain ANCOVAs to test the effect of the number of *APOE* ɛ4 alleles in the main text.

**Supplemental Results**

**Main and interaction effects of the *APOE* ɛ4 allele on RSFC**

The whole-brain ANCOVAs of RSFC had no significant main effects of *APOE* ɛ4 genotypes and no effects of the interaction between sex and the *APOE* ɛ4 allele on RSFC with right DLPFC as well as RSFC with bilateral hippocampi.

**Main and interaction effects of the *APOE* ɛ2 allele on non-whole-brain imaging metrics**

ANCOVAs have shown no significant main effects of the *APOE* ɛ2 allele for the entire unit or significant genotype–sex interaction effects on non-whole-brain imaging metrics. Using the uncorrected threshold, ANCOVAs showed (a) significant primary negative effects of the number of *APOE* ɛ2 alleles on mean rWMV for the second significant cluster below the right precentral gyrus (see main text) and (b) a significant *APOE* ɛ2 allele–sex interaction effect on digit span scores.

**Main and interaction effects of *APOE* ɛ2 allele in whole-brain analyses**

Whole-brain ANCOVAs have shown a significant negative main effect (regardless of sex) of the *APOE* ɛ2 allele on rWMV in the anatomical cluster that expanded across the following: cerebellar peduncle; genu and body of the corpus callosum; cerebral peduncle; areas of internal and external capsule; bilateral anterior, superior, and posterior corona radiate; left cingulum; left Heschl’s gyrus; bilateral stria terminalis; bilateral superior longitudinal fasciculus; bilateral superior fronto-occipital fasciculus; and right inferior fronto-occipital fasciculus.

There were no other significant results in whole-brain analyses.

**Supplemental discussion**

**Main and interaction effects of the *APOE* ɛ2 allele**

Non-whole-brain and whole-brain imaging analyses for assessing the effects of the *APOE* ɛ2 allele on neural structure revealed significant negative main effects on rWMV in widespread areas in the entire cohort but no significant sex interaction. These white matter areas with significant negative effects covered most of those with a significant *APOE* ɛ4 allele−sex interaction effect. Those in young individuals are not well investigated compared with the effects of the *APOE* ɛ2 allele in elderly individuals. Thus, the mechanisms that cause the observed effects of the *APOE* ɛ2 allele are not clear; however, we cite a few possibilities. *APOE* influences both synaptogenesis and synaptic plasticity, and its ɛ2 allele is involved in greater synaptic plasticity than the ɛ3 and ɛ4 alleles (Suri et al. 2013). Thus, low rWMV between subjects with the highest synaptic plasticity is consistent with the possible mechanism of the *APOE* ɛ4 allele discussed in the main text. It is still unclear why the *APOE* ɛ2 allele is not significantly associated with cognitive function, either alone or by interaction with sex. The *APOE* ɛ4 allele–sex interaction effects on Alzheimer’s disease are well established among elderly individuals, but the mechanisms that cause the protective effects of the *APOE* ɛ2 allele are not known. Nonetheless, based on a previous large-sample study, sex differences in these protective effects are minor or not found (Farrer et al. 1997). Moreover, the protective effect of the *APOE* ɛ2 allele against Alzheimer’s disease may not have an association with beta-amyloid deposition (Liu et al. 2013). Both the *APOE* ɛ2 and ɛ4 alleles have a relationship with increased cerebral amyloid angiopathy-related hemorrhage, which is a pathological condition in which amyloid spreads and deposits throughout the cerebral blood vessel walls (Biffi et al. 2010). Because amyloid deposition seldom occurs in young adults, the mechanisms that cause the effects of the *APOE* ɛ2 and ɛ4 alleles on neurocognition in young adults may not be the same as those in elderly individuals; however, this is highly speculative and future studies need to evaluate these problems.

**Supplemental Table 1**. Psychometric scale scores in each *APOE* ɛ2 allele and statistical values

|  | ɛ2/ɛ3  (male) | ɛ3/ɛ3  (male) | ɛ2/ɛ3  (female) | ɛ3/ɛ3 (female) | Main effect  P value^a^  (uncorrected, FDR) | Sex interaction effect^b^  (uncorrected, FDR) |
| --- | --- | --- | --- | --- | --- | --- |
| Digit span (N = 1001) | 37.65 ± 6.87 | 36.74 ± 6.98 | 32.63 ± 6.49 | 35.1 ± 6.37 | 0.318, 0.809 | 0.01, 0.173 |
| RAPM (N = 1005) | 29.04 ± 3.91 | 28.78 ± 3.75 | 28.63 ± 3.34 | 27.92 ± 3.94 | 0.435, 0.837 | 0.371, 0.809 |
| Total TBIT score (N = 912) | 116.3 ± 12.99 | 114.42 ± 12 | 110.62 ± 11.52 | 109.48 ± 11.45 | 0.385, 0.809 | 0.51, 0.842 |
| Perception factor of TBIT (N = 912) | 49.85 ± 7.68 | 49.49 ± 7.41 | 48.68 ± 6.74 | 48.97 ± 6.6 | 1, 1.05 | 1, 1.05 |
| Word-Color task (items) (N = 1002) | 72.16 ± 8.01 | 71.2 ± 8.11 | 69.55 ± 7.27 | 70.05 ± 7.29 | 1, 1.05 | 0.2, 0.726 |
| Color-Word task (items) (N = 1003) | 51.32 ± 6.91 | 52.53 ± 7.17 | 52.47 ± 6 | 52.81 ± 6.35 | 0.135, 0.726 | 0.961, 1.05 |
| Reverse Stroop task (items) (N = 1001) | 60.07 ± 7.48 | 59.94 ± 8.53 | 58.76 ± 7.28 | 59.61 ± 7.75 | 0.765, 1.05 | 0.163, 0.726 |
| Stroop task (items) (N = 1003) | 48.81 ± 6.71 | 48.31 ± 7.97 | 48.97 ± 6.26 | 49.72 ± 6.64 | 1, 1.05 | 0.298, 0.809 |
| S-A creativity test (N = 1005) | 36.58 ± 10.53 | 37.22 ± 10.6 | 39.42 ± 10.47 | 39.04 ± 9.62 | 1, 1.05 | 0.22, 0.726 |
| Significant cluster 1 of the effect of the *APOE* ɛ4 allele (N = 1005) | 0.6526 ± 0.0675 | 0.6649 ± 0.0724 | 0.5716 ± 0.0574 | 0.5869 ± 0.0581 | 0.124, 0.726 | 1, 1.05 |
| Significant cluster 2 of the effect of the *APOE* ɛ4 allele (N = 1005) | 0.2115 ± 0.0328 | 0.2239 ± 0.0358 | 0.1931 ± 0.0297 | 0.2000 ± 0.0320 | 0.015, 0.173 | 0.500, 0.842 |

FDR: False Discovery Rate, RAPM: Raven’s Advanced Progressive Matrix, TBIT: Tanaka B-type Intelligence Test

^a^P values for the main effects of the *APOE* ɛ2 allele

^b^P value for the interaction between sex and the *APOE* ɛ2 allele

**Supplemental Table 2**. Brain regions exhibiting significant main negative effects of one *APOE* ɛ2 allele

| No | Included large bundles** (number of significant voxels in the left and right side of each anatomical area) | x | y | z | TFCE value | Corrected *p* value (TFCE, FWE) | Cluster size (voxel) |
| --- | --- | --- | --- | --- | --- | --- | --- |
| 1 | The middle cerebellar peduncle (21)/genu of corpus callosum (74)/body of corpus callosum (84)/fornix (2)/cerebral peduncle (L:358, R:281)/anterior limb of internal capsule (L:886, R:808)/posterior limb of internal capsule (L:1194, R:1252)/retrolenticular part of internal capsule (L:213, R:347)/anterior corona radiata (L:497, R:858)/superior corona radiata (L:997, R:1248)/posterior corona radiata (L:124)/external capsule (L:596, R:526)/cingulum (L:238, R:1)/Heschl gyrus (L:238, R:1)/stria terminalis (L:77, R:155)/superior longitudinal fasciculus (L:180, R:733)/superior fronto-occipital fasciculus (L:163, R:138)/inferior fronto-occipital fasciculus (R:191) | 25.5 | 19.5 | 0 | 1655.29 | 0.013 | 35679 |

* The anatomical labels and significant clusters of major white matter fibers were determined using the ICBM DTI-81 Atlas (<http://www.loni.ucla.edu/>).

**Supplemental Figure legends**

**Supplemental Fig 1.** The significant negative main effects of one *APOE* ɛ2 allele between *APOE* ɛ3 allele carriers on rWMV. The upper panels indicate the areas of significant effects. The results were obtained by placing a threshold for TFCE of P < 0.05 based on 5000 permutations. Findings are covered on a “single-subject T1” image from SPM8. The color bar is the TFCE value. The lowest panel is a profile of rWMV in the significant cluster area for both sexes with ɛ2/ɛ2 and ɛ2/ɛ3 genotypes.

**Supplemental Fig 1.**


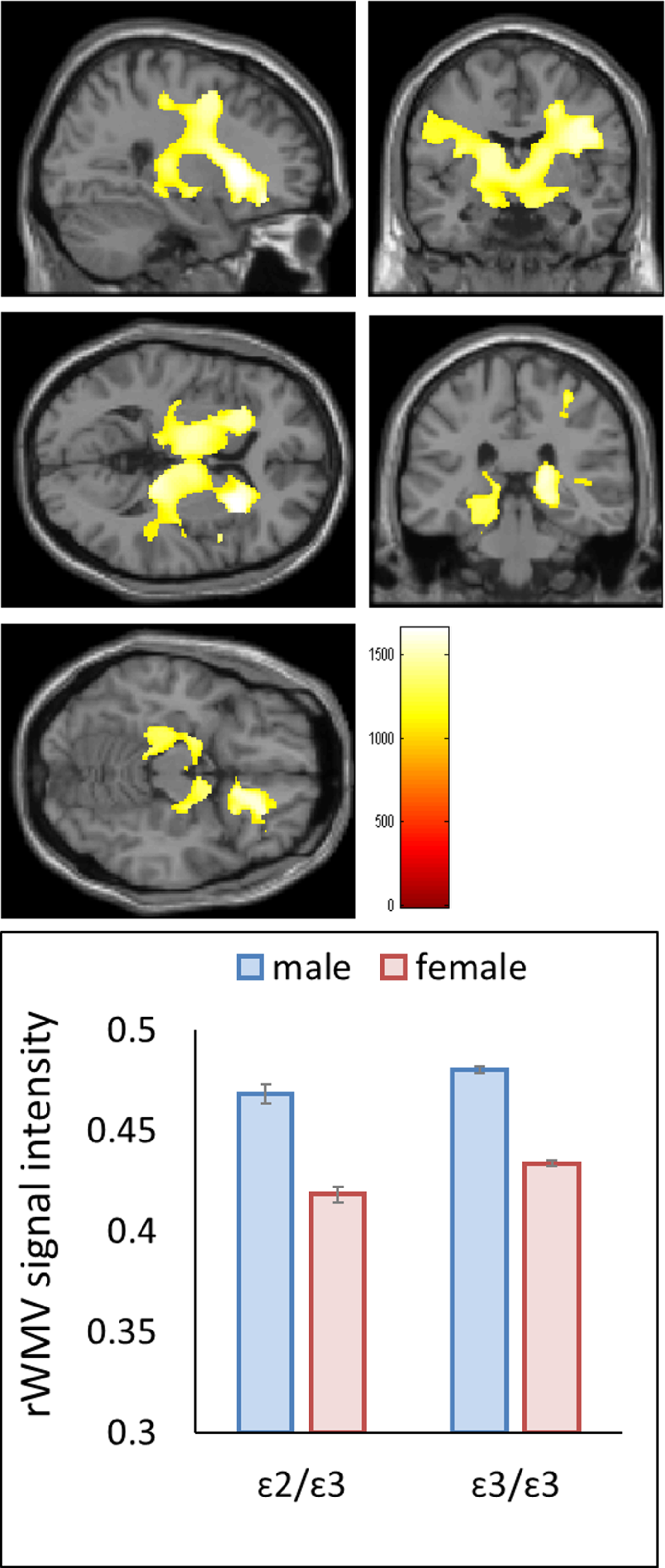


**References**

Acevedo SF, Piper BJ, Craytor MJ, Benice TS, Raber J. 2010. Apolipoprotein E4 and sex affect neurobehavioral performance in primary school children. Pediatr Res. 67: 293.

Biffi A, Sonni A, Anderson CD, Kissela B, Jagiella JM, Schmidt H, Jimenez‐Conde J, Hansen BM, Fernandez‐Cadenas I, Cortellini L. 2010. Variants at APOE influence risk of deep and lobar intracerebral hemorrhage. Ann Neurol. 68: 934-943.

Chao-Gan Y, Brian C, Clare K, Stan C, R Cameron C, Adriana Di M, Qingyang L, Xi-Nian Z, F Xavier C, Michael P M. 2013. A comprehensive assessment of regional variation in the impact of head micromovements on functional connectomics. Neuroimage. 76: 183-201.

Cherbuin N, Leach LS, Christensen H, Anstey KJ. 2007. Neuroimaging and APOE genotype: a systematic qualitative review. Dement Geriatr Cogn Disord. 24: 348-362.

Damoiseaux JS, Rombouts S, Barkhof F, Scheltens P, Stam CJ, Smith SM, Beckmann CF. 2006. Consistent resting-state networks across healthy subjects. Proceedings of the National Academy of Sciences. 103: 13848-13853.

Farrer LA, Cupples LA, Haines JL, Hyman B, Kukull WA, Mayeux R, Myers RH, Pericak-Vance MA, Risch N, Van Duijn CM. 1997. Effects of age, sex, and ethnicity on the association between apolipoprotein E genotype and Alzheimer disease: a meta-analysis. JAMA. 278: 1349-1356.

Friston KJ, Williams S, Howard R, Frackowiak RS, Turner R. 1996. Movement-related effects in fMRI time-series. Magn Reson Med. 35: 346-355.

Greicius MD, Krasnow B, Reiss AL, Menon V. 2003. Functional connectivity in the resting brain: a network analysis of the default mode hypothesis. Proc Natl Acad Sci U S A. 100: 253-258.

Heise V, Filippini N, Ebmeier K, Mackay C. 2011. The APOE ɛ4 allele modulates brain white matter integrity in healthy adults. Mol Psychiatry. 16: 908-916.

Liu C-C, Kanekiyo T, Xu H, Bu G. 2013. Apolipoprotein E and Alzheimer disease: risk, mechanisms and therapy. Nature Reviews Neurology. 9: 106-119.

Neu SC, Pa J, Kukull W, Beekly D, Kuzma A, Gangadharan P, Wang L-S, Romero K, Arneric SP, Redolfi A. 2017. Apolipoprotein E genotype and sex risk factors for Alzheimer disease: a meta-analysis. JAMA neurology. 74: 1178-1189.

Oldfield RC. 1971. The assessment and analysis of handedness: the Edinburgh inventory. Neuropsychologia. 9: 97-113.

Power JD, Barnes KA, Snyder AZ, Schlaggar BL, Petersen SE. 2012. Spurious but systematic correlations in functional connectivity MRI networks arise from subject motion. Neuroimage. 59: 2142-2154.

Shaw P, Lerch JP, Pruessner JC, Taylor KN, Rose AB, Greenstein D, Clasen L, Evans A, Rapoport JL, Giedd JN. 2007. Cortical morphology in children and adolescents with different apolipoprotein E gene polymorphisms: an observational study. The Lancet Neurology. 6: 494-500.

Song M, Zhou Y, Li J, Liu Y, Tian L, Yu C, Jiang T. 2008. Brain spontaneous functional connectivity and intelligence. Neuroimage. 41: 1168-1176.

Suri S, Heise V, Trachtenberg AJ, Mackay CE. 2013. The forgotten APOE allele: a review of the evidence and suggested mechanisms for the protective effect of APOE ɛ2. Neurosci Biobehav Rev. 37: 2878-2886.

Takeuchi H, Taki Y, Nouchi R, Hashizume H, Sekiguchi A, Kotozaki Y, Nakagawa S, Miyauchi CM, Sassa Y, Kawashima R. 2014a. Effects of Multitasking-Training on Gray Matter Structure and Resting State Neural Mechanisms. Hum Brain Mapp. 35: 3646-3660.

Takeuchi H, Taki Y, Nouchi R, Sekiguchi A, Hashizume H, Sassa Y, Kotozaki Y, Miyauchi CM, Yokoyama R, Iizuka K. 2014b. Association between resting-state functional connectivity and empathizing/systemizing. Neuroimage. 99: 312-322.

Takeuchi H, Taki Y, Nouchi R, Sekiguchi A, Hashizume H, Sassa Y, Kotozaki Y, Miyauchi CM, Yokoyama R, Iizuka K, Seishu N, Tomomi N, Kunitoki K, Kawashima R. 2015. Degree centrality and fractional amplitude of low-frequency oscillations associated with Stroop interference. Neuroimage. 119: 197-209.

Takeuchi H, Taki Y, Nouchi R, Yokoyama R, Kotozaki Y, Nakagawa S, Sekiguchi A, Iizuka K, Yamamoto Y, Hanawa S, Araki T, Miyauchi CM, Shinada T, Sakaki K, Nozawa T, Ikeda S, Yokota S, Daniele M, Sassa Y, Kawashima R. 2017. Regional homogeneity, resting-state functional connectivity and amplitude of low frequency fluctuation associated with creativity measured by divergent thinking in a sex-specific manner. Neuroimage. 152.

Takeuchi H, Taki Y, Sassa Y, Hashizume H, Sekiguchi A, Fukushima A, Kawashima R. 2011. Verbal working memory performance correlates with regional white matter structures in the fronto-parietal regions. Neuropsychologia. 49: 3466-3473

Takeuchi H, Taki Y, Thyreau B, Sassa Y, Hashizume H, Sekiguchi A, Nagase T, Nouchi R, Fukushima A, Kawashima R. 2013. White matter structures associated with empathizing and systemizing in young adults. Neuroimage. 77: 222-236.

Tanizaki H. 2006. On small sample properties of permutation tests: A significance test for regression models. Kobe University Economic Review. 52: 27-40.

Yan C-G, Cheung B, Kelly C, Colcombe S, Craddock RC, Martino AD, Li Q, Zuo X-N, Castellanos FX, Milham MP. 2013. A comprehensive assessment of regional variation in the impact of head micromovements on functional connectomics. NeuroImage. 76: 183-201.

Yan C-G, Wang X-D, Zuo X-N, Zang Y-F. 2016. DPABI: Data Processing & Analysis for (Resting-State) Brain Imaging. Neuroinformatics. 1-13.
